# Macrophage-derived apoptotic vesicles regulate fate commitment of mesenchymal stem cells via miR155

**DOI:** 10.1186/s13287-022-03004-w

**Published:** 2022-07-16

**Authors:** Yuan Zhu, Xiao Zhang, Kunkun Yang, Yuzi Shao, Ranli Gu, Xuenan Liu, Hao Liu, Yunsong Liu, Yongsheng Zhou

**Affiliations:** 1 Department of Prosthodontics, Peking University School and Hospital of Stomatology, 22 Zhongguancun South Avenue, Beijing, 100081 China; 2 The Central Laboratory, Peking University School and Hospital of Stomatology, 22 Zhongguancun South Avenue, Beijing, 100081 China; 3 National Center of Stomatology, National Clinical Research Center for Oral Diseases, National Engineering Research Center of Oral Biomaterials and Digital Medical Devices, Beijing Key Laboratory of Digital Stomatology, Research Center of Engineering and Technology for Computerized Dentistry Ministry of Health, Peking University School and Hospital of Stomatology, Beijing, 100081 China

**Keywords:** Macrophage, Apoptotic vesicles, Adipogenesis, Osteogenesis, MSCs, microRNA155

## Abstract

**Background:**

In tissue engineering, mesenchymal stem cells (MSCs) are common seed cells because of abundant sources, strong proliferation ability and immunomodulatory function. Numerous researches have demonstrated that MSC-macrophage crosstalk played a key role in the tissue engineering. Macrophages could regulate the differentiation of MSCs via different molecular mechanisms, including extracellular vesicles. Apoptotic macrophages could generate large amounts of apoptotic vesicles (apoVs). ApoVs are rich in proteins, RNA (microRNAs, mRNAs, ncRNAs, etc.) and lipids, and are a key intercellular communication mediator that can exert different regulatory effects on recipient cells. MiRNAs account for about half of the total RNAs of extracellular vesicles, and play important roles in biological processes such as cell proliferation and differentiation, whereas the functions of macrophage-derived apoVs remain largely unknown. There was no research to clarify the role of macrophage-derived apoVs in MSC fate choices. In this study, we aimed to characterize macrophage-derived apoVs, and investigate the roles of macrophage-derived apoVs in the fate commitment of MSCs.

**Methods:**

We characterized macrophage-derived apoVs, and investigated their role in MSC osteogenesis and adipogenesis in vitro and in vivo. Furthermore, we performed microRNA loss- and gain-of-function experiments and western blot to determine the molecular mechanism.

**Results:**

Macrophages could produce a large number of apoVs after apoptosis. MSCs could uptake apoVs. Then, we found that macrophage-derived apoVs inhibited osteogenesis and promoted adipogenesis of MSCs in vitro and in vivo. In mechanism, apoVs were enriched for microRNA155 (miR155), and apoVs regulated osteogenesis and adipogenesis of MSCs by delivering miR155. Besides, miR155 regulated osteogenesis and adipogenesis of MSCs cultured with macrophage-derived apoVs via the SMAD2 signaling pathway.

**Conclusions:**

Macrophage-derived apoVs could regulate the osteogenesis and adipogenesis of MSCs through delivering miR155, which provided novel insights for MSC-mediated tissue engineering.

**Supplementary Information:**

The online version contains supplementary material available at 10.1186/s13287-022-03004-w.

## Background

Over the past several decades, tissue engineering has been an important research direction to repair defects in regenerative medicine [1–5]. It involves three crucial components: seed cells, scaffolds, and bioactive molecules [6–8]. Seed cells should have self-renewal ability and differentiation potential, be able to establish stable cell lines, and have the lowest possible antigenicity [9–11]. MSCs have been widely applied in regenerative medicine research because of their abundance, strong proliferation ability and immunomodulatory function [12–16].

Macrophages are important modulators of host defense. Under physiological or pathological conditions, resting macrophages (M0) can polarize into proinflammatory phenotype (M1) and anti-inflammatory phenotype (M2) [17–20]. Many investigations have indicated that MSC-macrophage crosstalk occupied an important role in the osteogenic differentiation of MSCs through different molecular mechanisms, especially through extracellular vehicles (EVs) [21–23]. Kang et al. reported that M0 and M2 EVs could promote bone regeneration, while M1 EVs could inhibit bone regeneration [21]. Xiong et al. showed that exosomes secreted by M2 macrophages promoted osteogenesis, whereas exosomes secreted by M1 macrophages inhibited osteogenesis of bone mesenchymal stem cells [22]. Exosomes derived from M1 macrophages significantly promoted the expression of PPAR-γ and lipid droplet formation [23]. Therefore, vesicles produced by macrophages in different polarization states have different effects on MSCs.

Macrophages can secrete vesicles in a physiological state, they can also secrete many apoptotic vesicles (apoVs) under an apoptotic state, but little is known about the macrophage-derived apoVs. Apoptosis is the spontaneous and orderly death of cells and contributes to the maintenance of homeostasis [24–26]. ApoVs are rich in protein, RNAs and lipids, and they are a key intercellular communication medium [27]. Our previous study has investigated that functional efferocytosis of apoVs could restore liver macrophage homeostasis and ameliorates type 2 diabetes [28]. In 2018, Liu D et al. reported that apoptotic bodies could maintain bone homeostasis in physiological and pathological conditions and may have therapeutic potential for osteoporosis [29]. In 2020, Liu H et al. showed that extensive apoptosis occurred soon after stem cell transplantation, and a large number of apoptotic bodies were released, enhancing angiogenesis and having a therapeutic effect [30]. However, the regulation of macrophage-derived apoVs on fate choices of MSCs is unclear. In this study, we detected that macrophage-derived apoVs could promote adipogenesis and inhibit osteogenesis of MSCs both in vitro and in vivo, implicating miR155 in the mechanism by which apoVs regulate MSC adipogenesis and osteogenesis. Our study not only clarified the novel functions of macrophage-derived apoVs on differentiation of MSCs, but also gave hints for tissue engineering.

## Methods

### Culture of RAW 264.7 macrophages and MSCs

Primary RAW264.7 macrophages and hASCs were obtained from ScienCell Research Laboratories and these cells were grown in proliferation medium (PM) which contained 10% (*v/v*) fetal bovine serum (FBS) and antibiotics in DMEM.

### Osteogenic and adipogenic induction of MSCs

The osteogenic medium (OM) contained 10 nM dexamethasone, 200 µM ascorbic acid and 10 mM β-glycerophosphate in DMEM with 10% (*v/v*) FBS and antibiotics. The adipogenic medium (AM) contained 100 nM dexamethasone, 50 nM insulin, 0.5 mM 3-isobutyl-1-methylxanthine, and 200 µM indomecin in DMEM with 10% (*v/v*) FBS and antibiotics.

### TUNEL staining

Apoptosis was detected using the TUNEL Cell Apoptosis Detection Kit (Applygen). Briefly, normal or apoptotic macrophages were fixed and treated with 0.2% Triton X-100. Images were obtained using a fluorescence microscope (Olympus) after exposure to rhodamine fluorescein (red) labeled dUTP solution in the dark for 1 h.

### Isolation and purification of apoVs

ApoVs were collected as described previously with modifications [28]. When macrophages reached 80–90% confluence, serum-free medium containing 500 nM staurosporine (STS) (Enzo Life Sciences) was replaced and the supernatant was collected 12 h later. The supernatant was centrifuged at 800 g for 10 min and the precipitate was discarded, then, at 16,000 g for 30 min and the precipitate was collected and washed twice with filtered PBS. The Pierce BCA Protein Assay Kit (Thermo Scientific) was used to assess apoV concentration.

### Identification of apoVs

The morphology of the apoVs was observed by transmission electron microscopy (TEM). The apoVs were fixed and dropped onto a carbon-coated copper net. After air drying, apoVs were twice negatively stained with 1% uranyl acetate. Images were captured at 120 kV using an HT7700 TEM (Hitachi). The particle size of apoVs was determined by nanoparticle tracking analysis (NTA) using a Nano Sight NS300 (Malvern). After obtaining apoVs, RIPA lysate containing 2% protease inhibitor was added, and BCA protein quantitative test kit was used to detect the protein quantification of apoVs.

### ApoV uptake by MSCs

ApoVs were labeled with PKH-26 (Sigma-Aldrich) as described previously with modifications [31]. The labeled apoVs were washed in filtered PBS at 16,000 g for 30 min. Next, apoVs were incubated with MSCs for 4 and 8 h. After incubation, the cells were fixed. Then, the cells were treated with 0.1% Triton X-100 at room temperature for 7 min, washed twice with PBS. 5 µg/mL FITC-Cyclopeptide (Sigma-Aldrich) was added to stain F-actin. A solution of 6-diamidine-2-phenylindole (DAPI) was used to stain nuclei. The images were obtained using a LSM 5 EXCITER (Carl Zeiss).

### Oil red O staining and quantification

The MSCs were cultured in PM, AM, and AM with apoVs (AM + apoVs), respectively. The medium was changed every 2–3 days. After 14 days, oil red O staining and quantitative experiment were performed. Oil red O working solution was added to stain the cells. The staining was observed under the microscope at any time. For quantitative evaluation, stained cells were added to 100% isopropyl alcohol and dissolved for 10 min, the absorbance at 500 nm was measured.

### Alkaline phosphatase (ALP) staining and ALP activity, Alizarin red S (ARS) staining and quantification

The MSCs were divided into three groups: PM, OM, and OM added apoVs (OM + apoVs). The medium was changed every 2–3 days. After osteogenic induction for 7 days, ALP staining and activity assays were performed. BCIP / NBT staining kit (Beyotime Biotechnology) was used for ALP staining. ALP activity was quantified using an ALP Assay Kit (Nanjing Jiancheng) and the absorbance at 520 nm was measured. ALP activity (U/gprot) per gram of protein was calculated.

ARS staining and quantification were performed 14 days after osteogenic induction. Cells were incubated with Alizarin red buffer (Sigma-Aldrich). And 100 nM cetylpyridine solution was added for the quantification. The absorbance at 562 nm was measured.

### Real-time quantitative polymerase chain reaction (RT-qPCR)

Total RNA was extracted from cells using TRIzol reagent (Invitrogen) and cDNA was synthesized using a reverse transcription system (TaKaRa) RT-qPCR was performed on the ABI Prism 7500 real-time PCR System using SYBR Green Master Mix. *GAPDH* and *U6* were used as the reference genes. The primer sequences are listed in Table 1.

**Table 1:** List of primers used in this study

Gene	Forward primer (5′–3′)	Reverse primer (5′–3′)
*ALP*	GACCTCCTCGGAAGACACTC	TGAAGGGCTTCTTGTCTGTG
*BGLAP*	AGCAAAGGTGCAGCCTTTGT	GCGCCTGGGTCTCTTCACT
*RUNX2*	CCGCCTCAGTGATTTAGGGC	GGGTCTGTAATCTGACTCTGTCC
*PPARγ*	GAGGAGCCTAAGGTAAGGAG	GTCATTTCGTTAAAGGCTGA
*C/EBPα*	CGCAAGAGCCGAGATAAAGC	CACGGCTCAGCTGTTCCA
*GAPDH*	GAAGGTGAAGGTCGGAGTC	GAAGATGGTGATGGGATTTC
*mmu-miR155*	GCTTCGGTTAATGCTAATCGTG	CAGAGCAGGGTCCGAGGTA
*mmu-U6*	CGCTTCGGCAGCACATATAC	TTCACGAATTTGCGTGTCATC

### Western blot analysis

Cells were lysed in RIPA lysis buffer and protein extracts were subjected to 10% SDS-PAGE and transferred to polyvinylidene difluoride membranes. The membranes were incubated with the primary antibodies overnight and then with a peroxidase-conjugated secondary antibody. The ECL Kit (NCMbio) was used to detect protein bands.

### In vivo implantation of MSCs

The protocol was approved by the Institutional Animal Care and Use Committee of the Peking University Health Science Center (approval no. LA2021006). All surgeries were performed under anesthesia, and all efforts were made to minimize animal suffering.

The MSCs were cultured in (PM, AM, AM + apoVs) for 7 days, and mixed with the collagen membrane scaffolds. The number of cells inoculated in each tube was 1 × 10^6^, and the volume of the collagen membrane scaffold was about 8 mm × 8 mm × 2 mm. The mixtures (n = 10 per group) were implanted into BALB/c nude mice. The operation was carried out in an SPF animal operating room, 6-week-old female BALB/C nude mice were anesthetized, sterilized, and an incision of about 2 cm in length was made in the midline of the back, the subcutaneous implantation cavity was separated, and the cell-scaffold mixtures were implanted, sutured in position. The tissues were harvested after 6 weeks. All samples were performed by hematoxylin and eosin (H&E) staining and oil red O staining.

The MSCs were cultured in (PM, PM + apoVs), and mixed with β-TCP (RB-SK-005 G) (about 3 mm × 2 mm × 2 mm), and the number of cells inoculated in each tube was 1 × 10^6^. The tissues were harvested after 8 weeks. All samples were performed by H&E staining and Masson staining.

### MiRNA transient infection

Mimics-miR155 (miR155), mimics-negative control (miR-NC), inhibitor-miR155 (inhi-miR155), and inhibitor-negative control (inhi-NC) were purchased from Sangon Biotech. The sequences are listed in Table 2. When macrophages reached 60–70% density, miRNAs were transfected into macrophages using Lipofectamine 3000 (Invitrogen). After 48 h, cells were collected for RNA analysis. ApoVs were obtained as described above.

**Table 2:** List of miRNAs used in this study

	Sense (5′–3′)	Antisense (5′–3′)
*mmu-miR155*	UUAAUGCUAAUUGUGAUAGGGGU	CCCUAUCACAAUUAGCAUUAAUU
*mmu-mimics-NC*	UUGUACUACACAAAAGUACUG	GUACUUUUGUGUAGUACAAUU
*mmu-inhibitor-miR155*	ACCCCUAUCACAAUUAGCAUUAA	
*mmu-inhibitor-NC*	CAGUACUUUUGUGUAGUACAA	

### Statistical analysis

SPSS 19.0 software was used for statistical analysis. Comparisons of two groups were performed using independent two-tailed Student’s t tests, and comparisons of more than two groups by one-way ANOVA and Tukey’s post hoc test. Data were expressed as mean ± standard deviation. A value of *P* < 0.05 was considered indicative of statistical significance.

## Results

### Characterization of macrophage-derived apoVs and apoV uptake by MSCs.

**Fig. 1:**
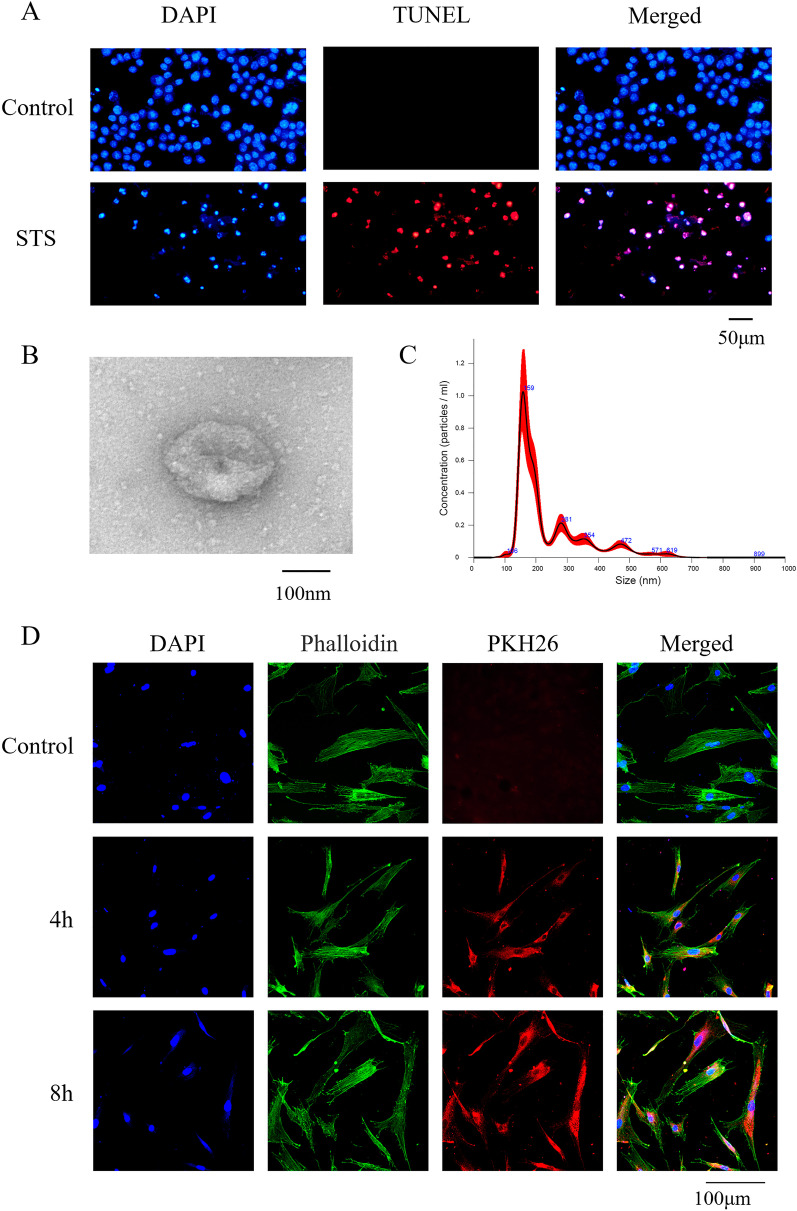
Characterization of macrophage-derived apoVs and apoVs uptake by MSCs. **A** TUNEL staining results in different groups. TUNEL positive stained cells were red. **B** Morphology of apoVs observed by TEM. **C** Particle size distribution of apoVs measured by NTA: the mean size ± SD of apoVs was 240.6 ± 115 nm. **D** MSCs were incubated with PKH-26-labeled apoVs (red) for 4 h and 8 h, respectively. The nuclei of MSCs were stained with DAPI (blue). The F-actin of MSCs was stained with phalloidin (green)

STS was used to induce apoptosis of macrophages. Apoptotic and normal macrophages were observed under the fluorescence microscope. A larger number of TUNEL positive stained cells (red) were observed in the STS group, while control group had seldom stained cells (Fig. 1A). Apoptotic macrophages could generate lots of apoVs; the output of apoVs was much higher than exosomes (Additional file 1: Fig. S1). TEM showed that apoVs had a cup-shaped morphology and the diameter was about 200 nm (Fig. 1B). NTA showed that the diameter distribution of apoVs was 240.6 ± 115 nm (Fig. 1C). To investigate whether apoVs could be ingested by MSCs, MSCs were cultured with PKH-26-labeled apoVs (red) for 4 h and 8 h, respectively. The nuclei of MSCs were stained with DAPI (blue). The F-actin of MSCs was stained with phalloidin (green). Confocal laser microscopy showed that red-stained particles appeared around the MSCs nucleus after 4 h, and the number of red-stained particles increased after 8 h (Fig. 1D).

### Macrophage-derived apoVs promoted adipogenesis of MSCs in vitro.

**Fig. 2:**
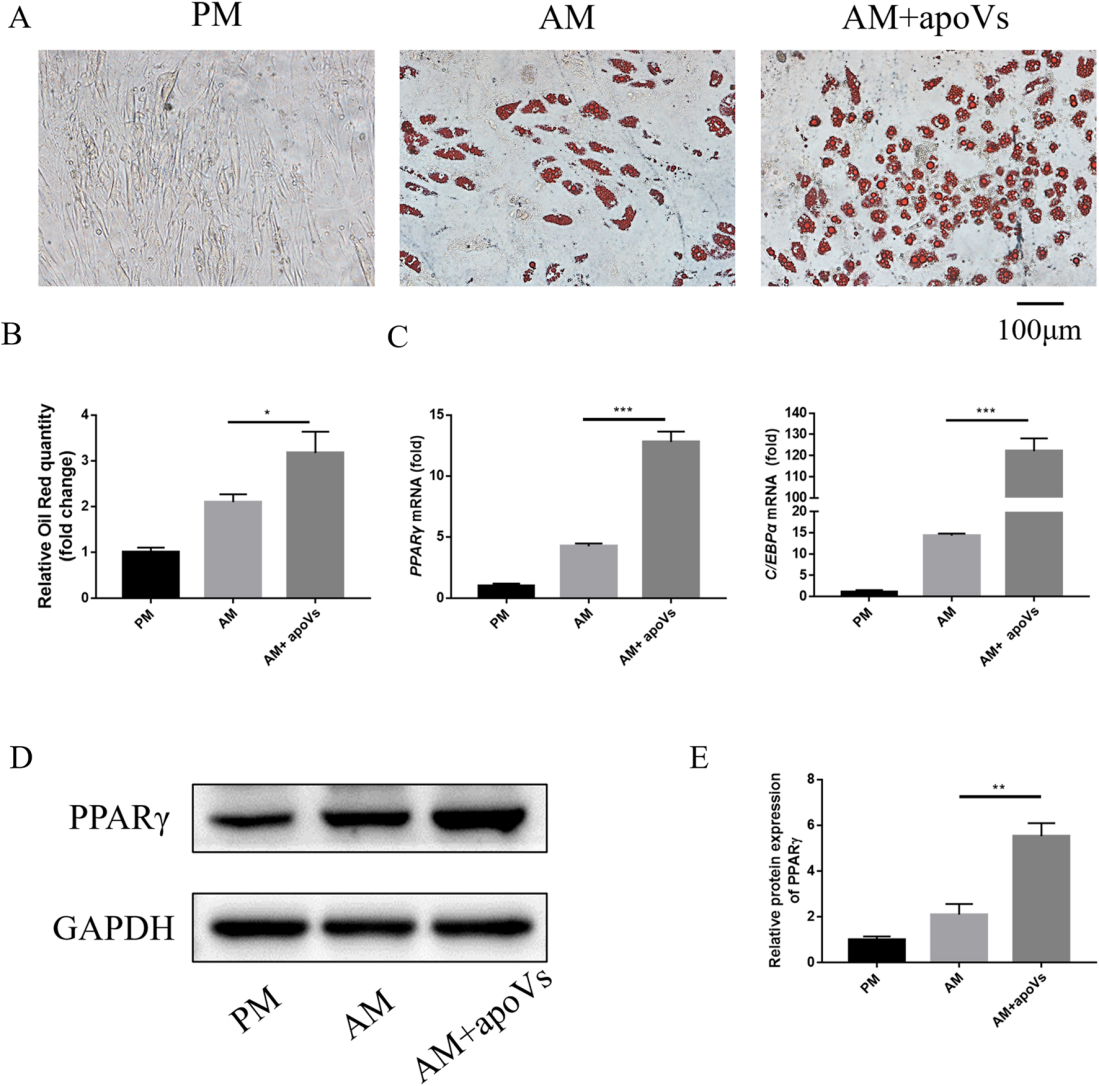
Macrophage-derived apoVs promoted adipogenesis of MSCs in vitro. **A** Macrophage-derived apoVs promoted adipogenesis as indicated by oil red O staining. **B** Oil red O quantitative analysis showed that apoVs promoted adipogenesis. **C** Macrophage-derived apoVs enhanced the mRNA expression of *PPARγ* and *C/EBPα* detected by RT-qPCR. **D** Western blot showed that the protein expression of PPARγ was up-regulated in the group treated with apoVs compared with the group without apoVs. **E** Western blot quantification showed the same result as Fig. 2D. *P < 0.05, **P < 0.01, ***P < 0.001

We set up different concentration gradients (0.5, 1, 2, 4 μg/mL) to clarify the optimal concentration of apoVs, and cultured MSCs in OM and AM, respectively, for 7 days. PPARγ was an important marker to measure the adipogenic differentiation of stem cells [32]; RUNX2 was an important marker to measure the osteogenic differentiation of stem cells [33]. In AM culture, 2 μg/ml apoVs significantly increased the *PPARγ* expression; in OM culture, 2 and 4 μg / ml apoVs inhibited the *RUNX2* expression levels more significantly (Additional file 2: Fig. S2). Therefore, we used 2 μg / mL apoVs in the subsequent experiments. Next, to further investigate the role of macrophage-derived apoVs in MSC adipogenesis, we treated MSCs under AM with or without apoVs. After 14 days, the cells were stained with oil red O. A great number of red-stained lipid droplets were formed when cells were cultured in AM, while the lipid droplets were significantly increased in MSCs treated with apoVs (Fig. 2A). In AM, oil red O quantitative analysis (Fig. 2B) of the group under stimulation by apoVs was higher than the group without apoVs (*P* < 0.05). PPARγ and C/EBPα were important markers to measure the adipogenic differentiation of stem cells [32, 34]. In addition, *PPARγ* and *C/EBPα* expression levels of the group treated with apoVs were significantly higher than group without apoVs (*P* < 0.001) (Fig. 2C). Moreover, the protein expression of PPARγ was up-regulated during adipogenesis in the group treated with apoVs compared with the group without apoVs (Fig. 2D, E).

### Macrophage-derived apoVs inhibited osteogenesis of MSCs in vitro.

**Fig. 3:**
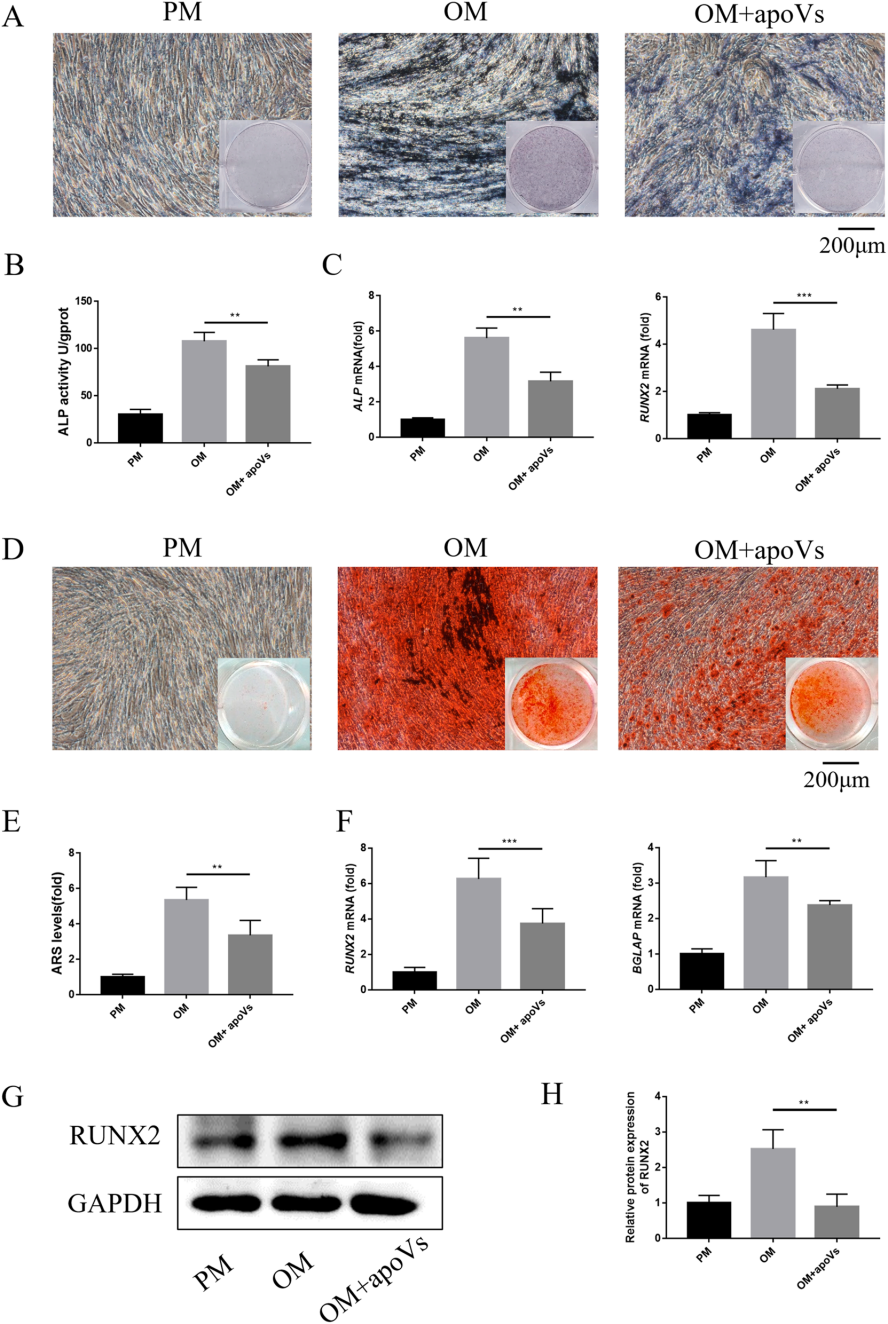
Macrophage-derived apoVs inhibited osteogenesis of MSCs in vitro. **A** ApoVs inhibited osteogenic differentiation as indicated by ALP staining. **B** ApoVs inhibited osteogenic differentiation as indicated by ALP quantification. **C** ApoVs inhibited the mRNA expression of *ALP* and *RUNX2* detected by RT-qPCR. **D** ApoVs inhibited osteogenesis as indicated by ARS staining. **E** ApoVs inhibited osteogenesis as indicated by ARS quantification. **F** ApoVs reduced the mRNA expression of *RUNX2* and *BGLAP* detected by RT-qPCR. **G** Western blot showed that the protein expression of RUNX2 was down-regulated in the group treated with apoVs compared with the group without apoVs. **H** Western blot quantification showed the same result as Fig. 3G. **P < 0.01, ***P < 0.001

To further explore the effect of macrophage-derived apoVs on osteogenesis of MSCs, we treated MSCs under OM with or without apoVs for 7 days. The cells were examined by ALP staining (Fig. 3A) and ALP quantification (Fig. 3B), the results showed that apoVs significantly inhibited osteogenic differentiation of MSCs. ALP, RUNX2 and BGLAP were important markers to measure the osteogenic differentiation of stem cells [33, 35, 36]. The expression of *ALP* and *RUNX2* was significantly decreased by apoVs (Fig. 3C). After treated MSCs under OM with or without apoVs for 14 days, the ARS staining and quantification showed similar results that apoVs could inhibit osteogenic differentiation of MSCs (Fig. 3D, E). Moreover, *RUNX2* and *BGLAP* expression was significantly decreased by treatment with apoVs for 14 days in OM (Fig. 3F). In addition, the protein expression of RUNX2 was down-regulated during osteogenesis by apoVs (Fig. 3G, H).

### Macrophage-derived apoVs promoted adipogenesis of MSCs in vivo.

**Fig. 4:**
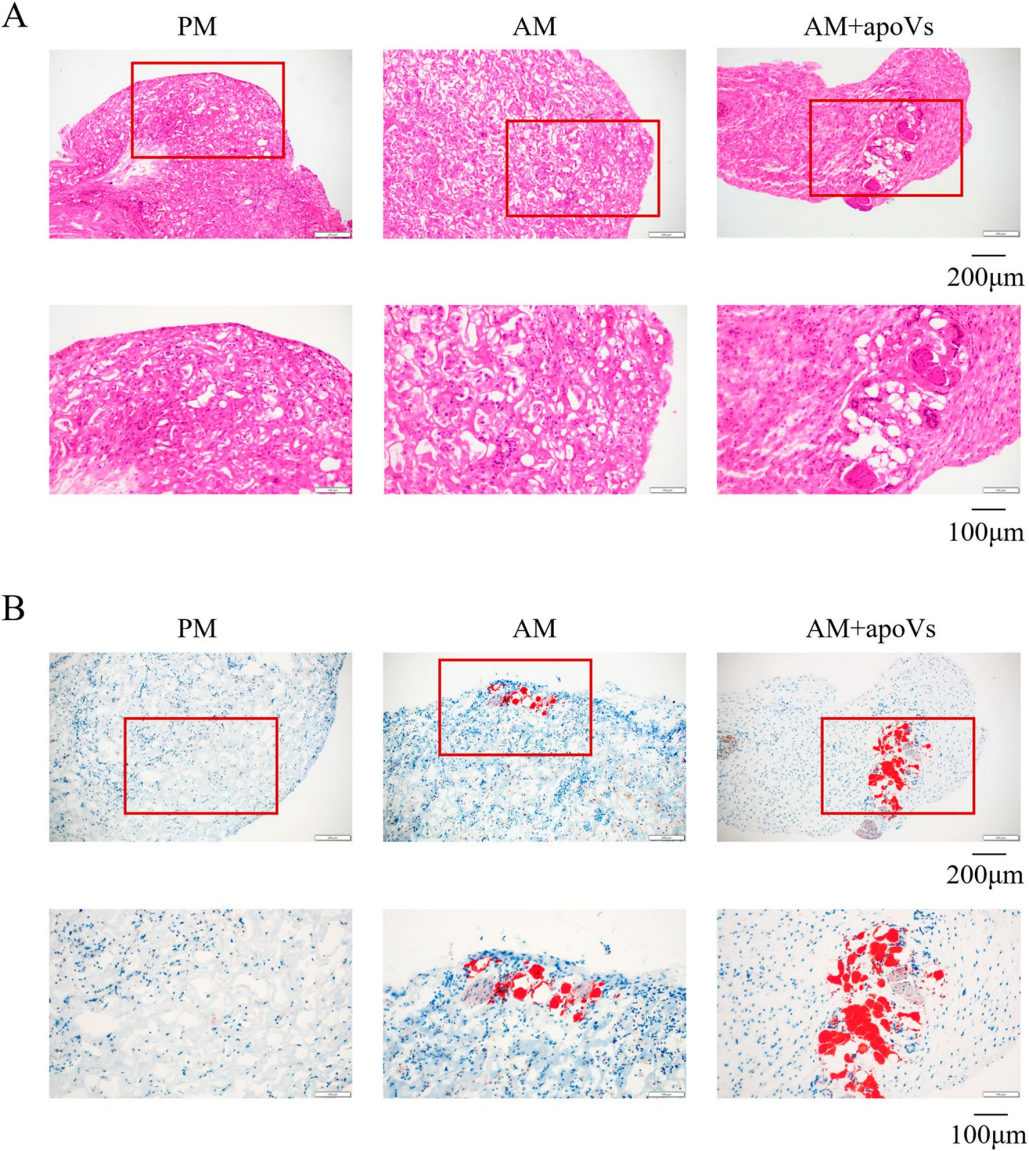
Macrophage-derived apoVs promoted adipogenesis of MSCs in vivo. **A** H&E staining of the PM, AM, and AM + apoVs groups of MSCs. **B** Oil red O staining of the PM, AM, and AM + apoVs groups of MSCs. The red rectangles indicated the corresponding magnified areas

To examine the role of macrophage-derived apoVs in MSC adipogenic differentiation in vivo, we combined MSCs (PM, AM, and AM + apoVs) with collagen sponges and implanted them into nude mice. H&E staining showed that the AM + apoVs group had more adipose tissue-like structures than the AM group, whereas the PM group showed a large amount of collagen membrane scaffold and no adipose tissue-like structures (Fig. 4A). Oil red O staining showed that the AM + apoVs group had more red-stained adipose tissue-like structures than the AM group (Fig. 4B). Therefore, macrophage-derived apoVs could promote adipogenesis of MSCs in vivo.

### Macrophage-derived apoVs inhibited osteogenesis of MSCs in vivo.

**Fig. 5:**
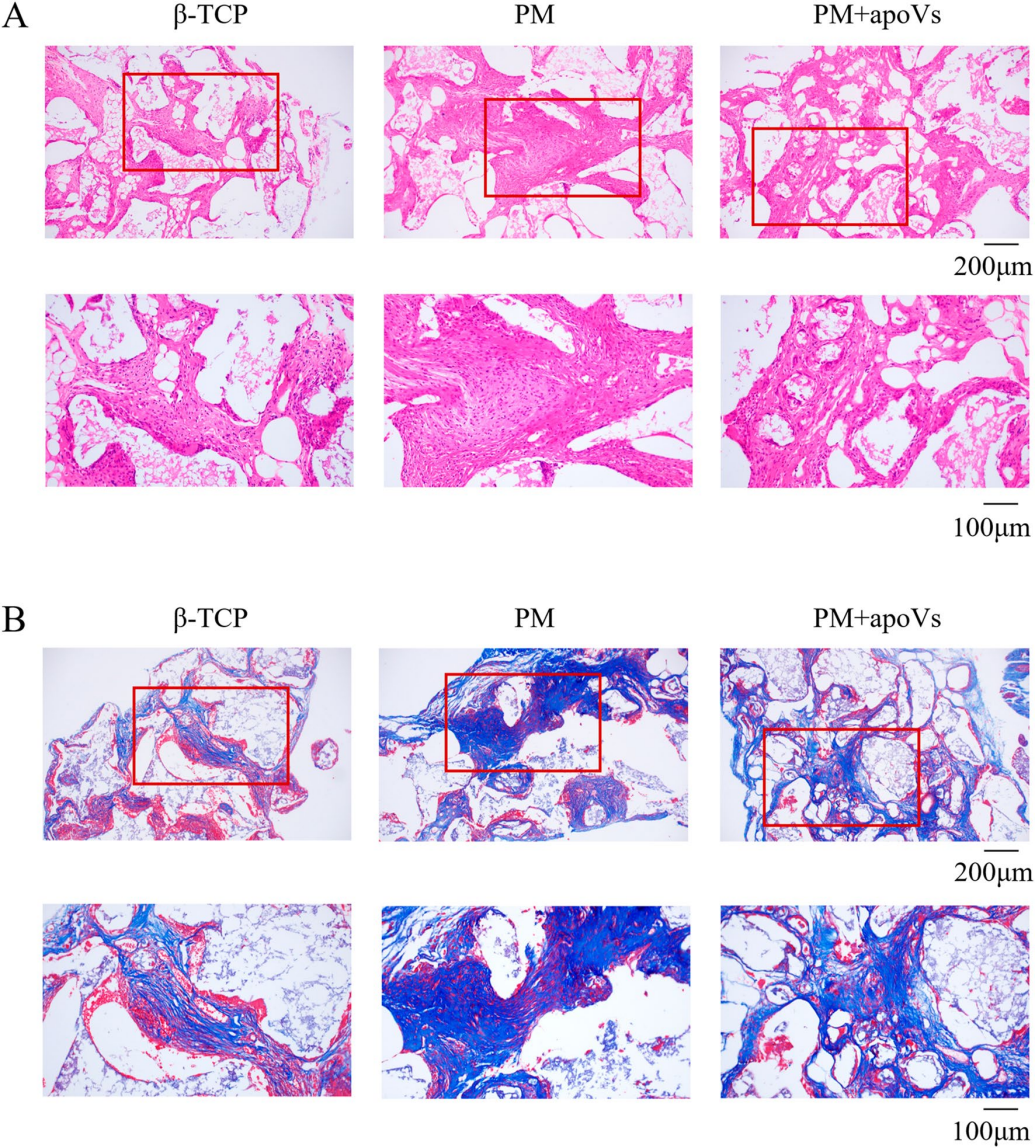
Macrophage-derived apoVs inhibited osteogenesis of MSCs in vivo. **A** H&E staining of the β-TCP, PM and PM + apoVs groups. **B** Masson staining of the β-TCP, PM and PM + apoVs groups. The red rectangles indicated the corresponding magnified areas

In order to determine the role of macrophage-derived apoVs in MSC osteogenesis in vivo, we mixed MSCs cultured in (PM and PM + apoVs) with β-TCP, then implanted them into nude mice. H&E staining (Fig. 5A) showed that the PM group had more new, strongly eosinophilic tissue compared to the β-TCP group, whereas the PM + apoVs group had fewer bone tissue-like structures than PM group. Masson staining (Fig. 5B) showed more blue-green collagen fibers in the PM group than the β-TCP group, whereas the PM + apoVs group had fewer blue-green collagen fibers than the PM group. Therefore, macrophage-derived apoVs inhibited osteogenesis of MSCs in vivo.

### MiR155 promoted adipogenesis of MSCs cultured with macrophage-derived apoVs.

**Fig. 6:**
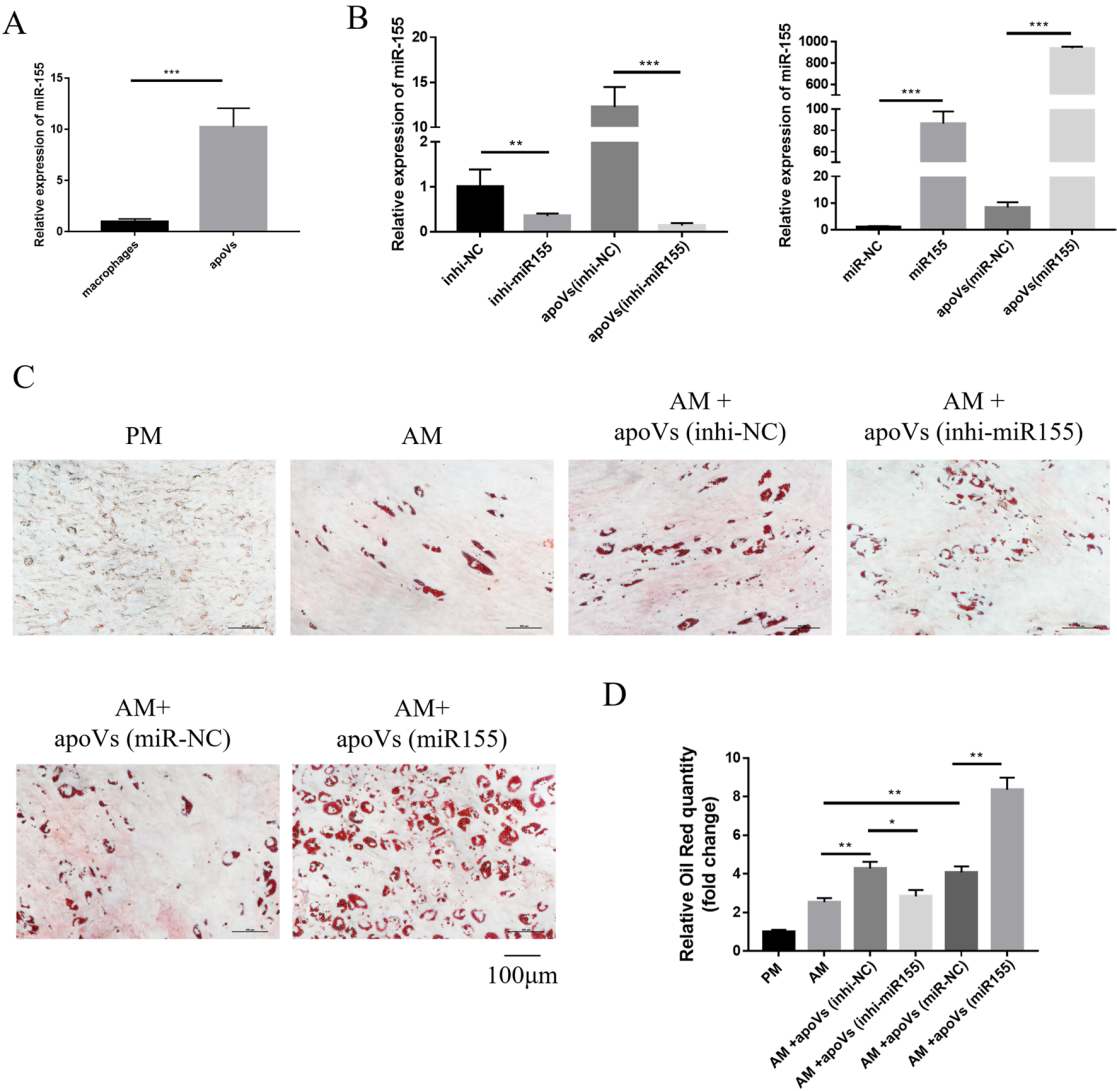
MiR155 promoted adipogenesis of MSCs cultured with macrophage-derived apoVs. **A** Expression levels of miR155 in macrophages and apoVs indicated that miR155 was highly enriched in apoVs. **B** Expression levels of miR155 in macrophages transfected with inhi-NC, inhi-miR155, miR-NC or miR155 and corresponding apoVs. **C** MSCs cultured in PM or AM treated with or without apoVs stained for oil red O. **D** Quantification of oil red O. *P < 0.05, **P < 0.01, ***P < 0.001

**Fig. 7:**
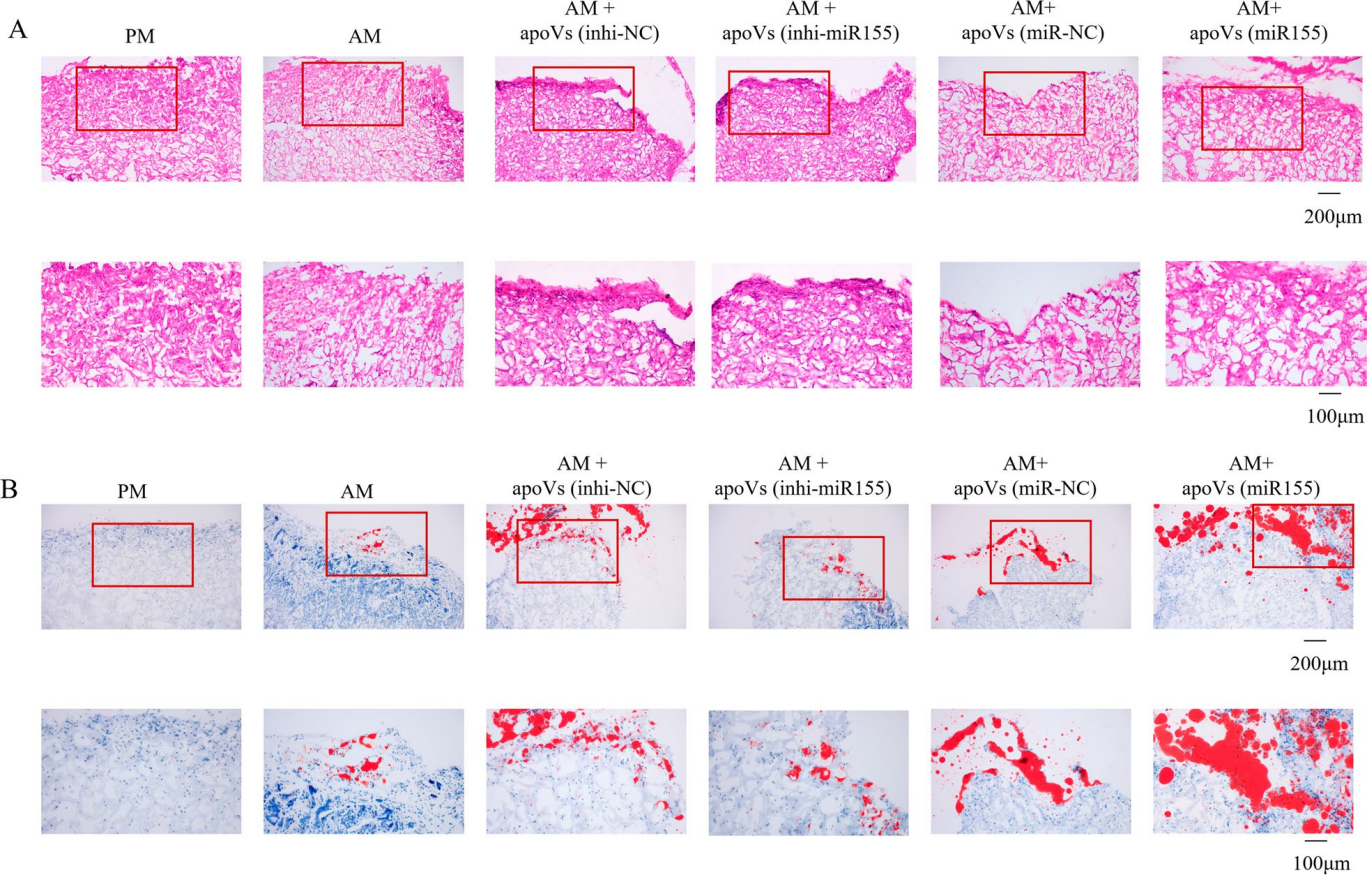
Macrophage-derived apoVs promoted adipogenesis of MSCs via miR155 in vivo. **A** H&E staining. **B** Oil red O staining. The red rectangles indicated the corresponding magnified areas

Several studies have shown that the contents of EVs including mRNAs, miRNAs, ncRNAs, protein and lipids. MiRNAs account for about half of the total RNAs of EVs, and play a key role in the transfer of biomolecules to recipient cells and cell-to-cell communication [37–39]. The content of apoVs was higher than that of exosomes, and the content of miRNA in apoVs was much higher than that of exosomes (Additional file 3: Fig. S3). We detected some key miRNAs involved in osteogenesis and adipogenesis processes, the variation of miR155 was most significant (Additional file 4: Fig. S4), so we mainly focused on miR155. Subsequently, we detected the expression of miR155 in the apoVs (Fig. 6A), and the results showed that miR155 was highly enriched in apoVs, so we mainly studied miR155 in the follow-up research. Next, we transfected macrophages with inhibitor-negative control (inhi-NC), inhibitor-miR155 (inhi-miR155), mimics-negative control (miR-NC) and mimics-miR155 (miR155), the expression levels of miR155 could be significantly decreased by (inhi-miR155) or increased by (miR155) in macrophages, and miR155 expression levels decreased or increased more significantly in corresponding apoVs: apoVs (inhi-NC), apoVs (inhi-miR155), apoVs (miR-NC) and apoVs (miR155), respectively (Fig. 6B). Subsequently, apoVs (inhi-NC), apoVs (inhi-miR155), apoVs (miR-NC) and apoVs (miR155) were added to MSCs for adipogenic induction. The results showed that the adipogenesis of MSCs was decreased in apoVs (inhi-miR155) group compared to the apoVs (inhi-NC) group. In addition, the adipogenesis ability of MSCs was improved in the apoVs (miR155) group compared to the apoVs (miR-NC) group (Fig. 6C, D). In order to determine the effect of miR155 on adipogenic differentiation of MSCs in vivo, MSCs treated with apoVs (inhi-NC), apoVs (inhi-miR155), apoVs (miR-NC) and apoVs (miR155) were subjected to adipogenic induction and implanted into nude mice subcutaneously. All samples were performed by H&E staining and oil red O staining. H&E staining showed that the AM + apoVs (miR155) group had more adipose tissue-like structures than the AM + apoVs (miR-NC) group, whereas the AM + apoVs (inhi-miR155) group showed a large amount of collagen membrane scaffold and fewer adipose tissue-like structures than AM + apoVs (inhi-NC) group (Fig. 7A). Oil red O staining showed the same results (Fig. 7B). Therefore, macrophage-derived apoVs could promote adipogenesis of MSCs via miR155 in vivo. Collectively, these results showed that miR155 promoted adipogenic differentiation of MSCs cultured with macrophage-derived apoVs.

### MiR155 inhibited osteogenesis of MSCs cultured with macrophage-derived apoVs.

**Fig. 8:**
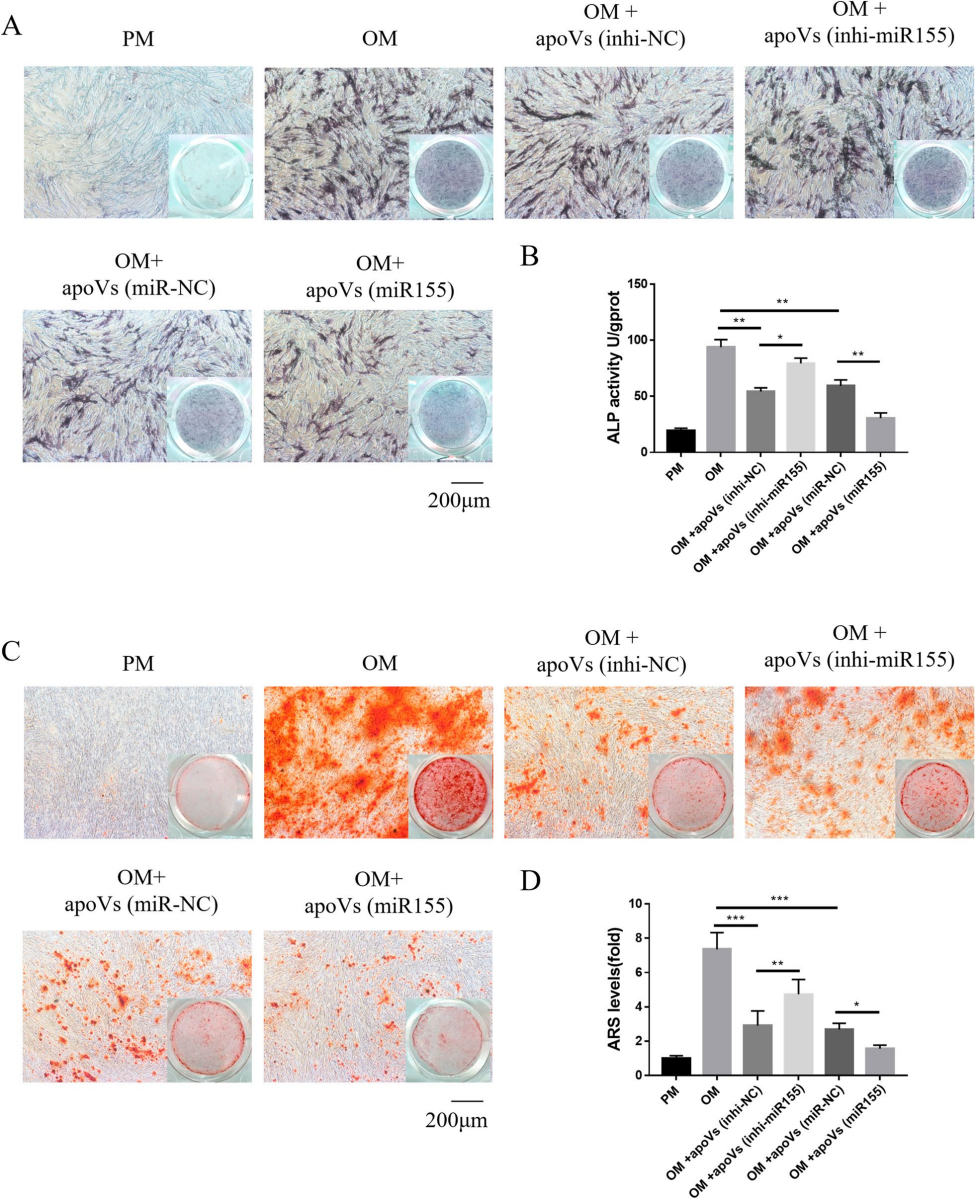
MiR155 inhibited osteogenesis of MSCs cultured with macrophage-derived apoVs. **A** MSCs cultured in PM or OM treated with or without apoVs stained for ALP. **B** Quantification of ALP. **C** MSCs cultured in PM or OM treated with or without apoVs stained for ARS. **D** Quantification of ARS. *P < 0.05, **P < 0.01, ***P < 0.001

In order to further clarify the role of miR155 in macrophage-derived apoVs in regulating the osteogenesis of MSCs, we cultured MSCs with apoVs (inhi-NC), apoVs (inhi-miR155), apoVs (miR-NC) and apoVs (miR155) in OM. The ALP staining and quantification showed that the osteogenesis of MSCs was up-regulated in the apoVs (inhi-miR155) group compared with apoVs (inhi-NC) group, while the osteogenesis of MSCs was decreased in apoVs (miR155) group than the apoVs (miR-NC) group (Fig. 8A, B). ARS staining and quantification confirmed these results (Fig. 8C, D). Therefore, our results suggested that miR155 could regulate osteogenesis and adipogenesis of MSCs in the presence of macrophage-derived apoVs.

### MiR155 regulated adipogenic and osteogenic differentiation of MSCs cultured with macrophage-derived apoVs via the SMAD2 signaling pathway.

**Fig. 9:**
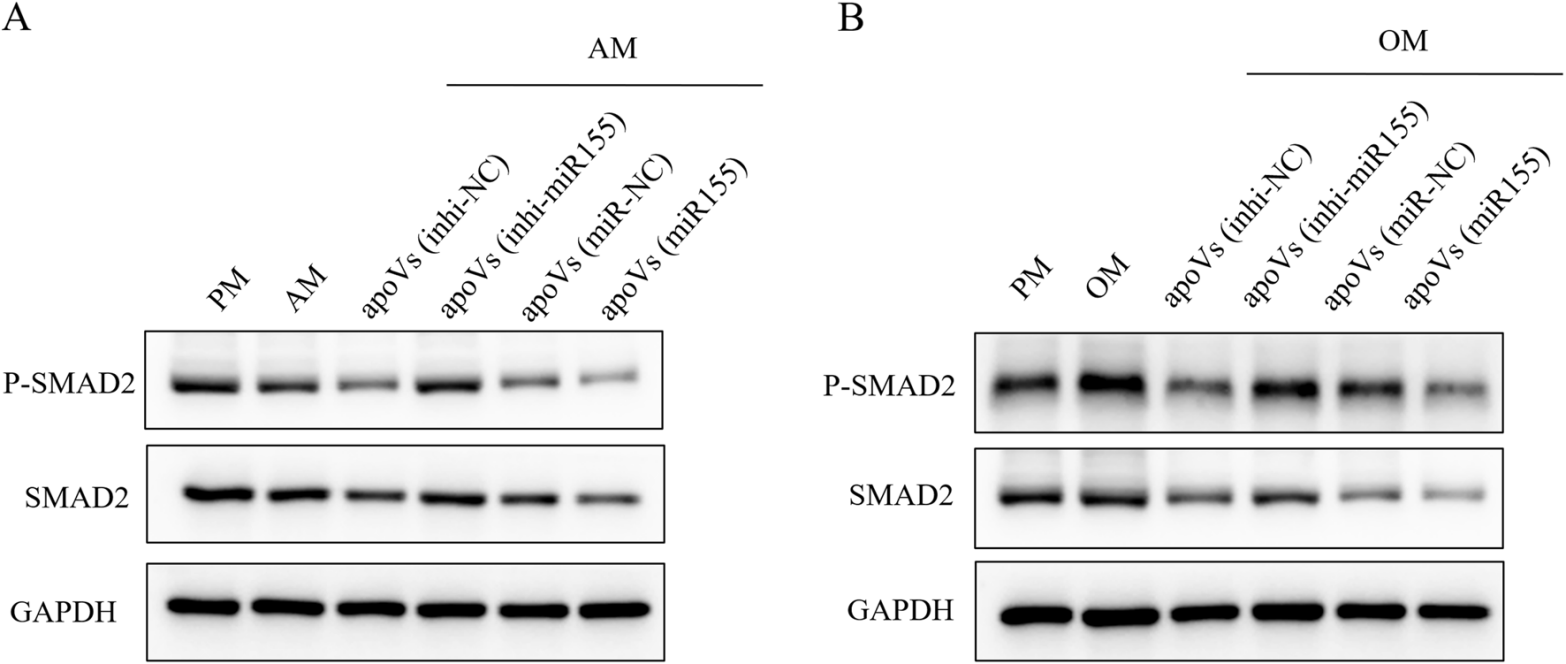
MiR155 regulated adipogenesis and osteogenesis of MSCs cultured with macrophage-derived apoVs via SMAD2 signaling pathway. **A** MiR155 inhibited the expression of SMAD2 and P-SMAD2 during the adipogenic differentiation of MSCs. **B** MiR155 inhibited the expression of SMAD2 and P-SMAD2 during the osteogenic differentiation of MSCs

**Fig. 10:**
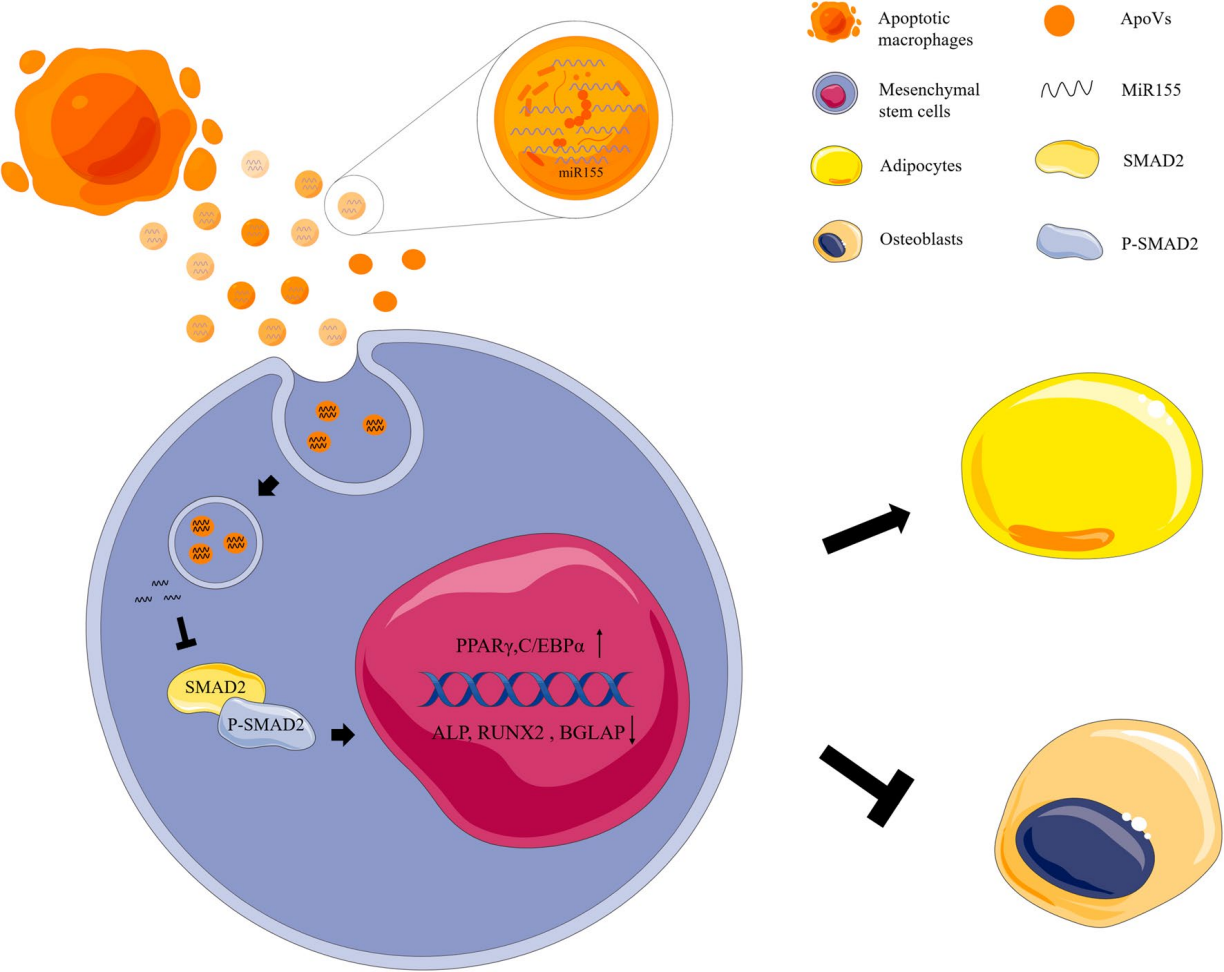
MiR155 regulated adipogenic and osteogenic differentiation of MSCs cultured with macrophage-derived apoVs via the SMAD2 signaling pathway

To elucidate the mechanism by which apoVs regulate stem cell differentiation, we screened key factors related to osteogenic and adipogenic differentiation, among which the down-regulated SMAD2 signaling pathway was more significant (Additional file 5: Fig. S5). The results indicated that apoVs could regulate the osteogenic and adipogenic differentiation of MSCs through the SMAD2 signaling pathway. Then, we focused on the SMAD2 signaling pathway. It has been reported that SMAD2 was a target gene of miR155 [40, 41], but the effect of macrophage-derived miR155 on the SMAD2 pathway of MSCs has not been investigated. So, we further studied the regulation of miR155 on the SMAD2 signaling pathway. Enhancement of the SMAD2 signaling pathway increased osteogenic differentiation and inhibited adipogenic differentiation [42–44]. We cultured MSCs with apoVs (inhi-NC), apoVs (inhi-miR155), apoVs (miR-NC) and apoVs (miR155) in AM and OM, then used western blot analysis to clarify the changes of SMAD2 pathway proteins (Fig. 9A, B). In the apoVs (inhi-miR155) group, the SMAD2 signaling pathway was up-regulated compared to the apoVs (inhi-NC) group. Moreover, the SMAD2 signaling pathway was down-regulated in the apoVs (miR155) group compared to the apoVs (miR-NC) group. Our data suggested that miR155 regulated adipogenic and osteogenic differentiation of MSCs cultured with macrophage-derived apoVs via the SMAD2 signaling pathway (Fig. 10).

## Discussion

Macrophages played important roles in stem cell survival and tissue repair [45]. Different polarization states of macrophages have different effects on stem cell differentiation. In 2018, He et al. reported that conditioned medium of M1 macrophages could promote the adipogenesis of bone marrow mesenchymal stem cells (BMMSCs), whereas conditioned medium of M0 and M2 macrophages could promote the osteogenesis of BMMSCs [46]. In 2020, Ma et al. showed that macrophages could inhibit the adipogenic differentiation of hASCs in vitro [47]. Furthermore, the stem cell–macrophage interaction was associated with the development of several adipose-related diseases [48–50]. These studies suggested that macrophages could regulate the differentiation of MSCs via different molecular mechanisms. However, the effect of macrophage-derived apoVs on the multidirectional differentiation of stem cells was unclear.

In this study, we detected the role of macrophage-derived apoVs in the fate commitment of MSCs. We investigated that apoptotic macrophages could secrete a large number of apoVs which had a cup-shaped morphology and the diameter was under 1 μm. The content of apoVs was higher than that of exosomes. ApoVs were ingested by MSCs. In addition, we detected that macrophage-derived apoVs could promote adipogenesis and inhibit osteogenesis of MSCs both in vitro and in vivo.

EVs secreted by cells contain a large number of miRNAs, which can be transported to recipient cells and change the expression of target genes [51–53]. Although specific miRNAs have no clear physiological functions, miRNAs are involved in almost all biological processes. In 2017, Gu et al. reported that miR155 inhibited osteogenesis by targeting the positive regulation of osteogenesis factors [54]. In 2019, Mao Z et al. identified that miR155 was highly expressed in osteoporotic patients and inhibited osteoclast activation and bone resorption [55]. Ying et al. found that miR155 was overexpressed in obese adipose tissue macrophage exosomes, and miR155-KO animals were insulin sensitive and glucose tolerant [56]. We found that apoVs were enriched in miR155. ApoVs could be taken up by MSCs, and the contents of the apoVs could be transferred to stem cells. In addition, miR155 regulated adipogenic and osteogenic differentiation of MSCs cultured with macrophage-derived apoVs via the SMAD2 signaling pathway. Therefore, macrophage-derived apoVs could regulate the osteogenesis and adipogenesis of MSCs through miR155, suggesting a novel tissue engineering method based on multidirectional differentiation of stem cells.

Soft tissue defect or depression caused by trauma, infection, operation or congenital malformation is very common in clinics. Adipose tissue engineering can repair soft tissue defects, correct contour deformation, and provide solutions for tissue reconstruction after soft tissue trauma [57–61]. Adipogenic differentiation of stem cells plays an important role in the construction of adipose tissue engineering and the treatment of soft tissue defects [62, 63]. Our results showed that macrophage-derived apoVs could promote stem cell adipogenesis through miR155, which suggested that apoVs could be used as miRNA carriers to treat soft tissue defects. Thus, macrophage-derived apoVs could provide a new soft tissue method.

Overall, our study still has some limitations. Further analysis of the binding of miR155 and SMAD2 by dual luciferase assay is needed. In future research, we plan to explore the influence of apoVs derived from M1 and M2 polarized macrophages on stem cell differentiation. Finally, further research is needed to enable the clinical application of apoVs in tissue engineering.

## Conclusion

Our study found that macrophage-derived apoVs could regulate osteogenesis and adipogenesis of MSCs through miR155. ApoVs could transport miRNAs and regulate multidirectional differentiation of stem cells, which provides a novel method for tissue engineering based on multidirectional differentiation of stem cells.

## Supplementary Information

**Additional file 1: Fig.S1**. Relative protein quantification of exosomes and apoVs. ***P < 0.001.

**Additional file 2: Fig. S2**. The *PPARγ* and *RUNX2* gene expression levels of MSCs treated with different concentration of macrophage-derived apoVs. *P < 0.05, **P < 0.01, ***P < 0.001 compared with AM or OM.

**Additional file 3: Fig. S3**. Relative expression of miR155 in the macrophage-derived exosomes and apoVs. ***P < 0.001.

**Additional file 4: Fig. S4**. Expression levels of different miRNAs in MSCs. *P < 0.05, **P < 0.01, ***P < 0.001 compared with AM.

**Additional file 5: Fig. S5**. Protein expression levels of different signaling pathways.

## Data Availability

The authors confirm that all data underlying the findings are fully available.

## Abbreviations

ALP: Alkaline phosphatase
AM: Adipogenic medium
ApoVs: Apoptotic vesicles
ARS: Alizarin Red S
BGLAP: Bone gamma-carboxyglutamic acid-containing protein
BMMSCs: Bone marrow mesenchymal stem cells
C/EBPα: CCAAT/enhancer-binding protein α
EVs: Extracellular vesicles
FBS: Fetal bovine serum
GAPDH: Glyceraldehyde3-phosphate dehydrogenase
hASCs: Human adipose-derived stem cells
H&E: Hematoxylin and eosin
inhi-miR155: Inhibitor-miR155
inhi-NC: Inhibitor-negative control
mimics: Mimics-miR155
miR155: MicroRNA155
miR-NC: Mimics-negative control
MSCs: Mesenchymal stem cells
NTA: Nanoparticle tracking analysis
OM: Osteogenic medium
PM: Proliferation medium
PPARγ: Peroxisome proliferator activated receptor γ
RT-qPCR: Real-time quantitative polymerase chain reaction
STS: Staurosporine
TEM: Transmission electron microscopy
