# Supplementary figures and images for: Macrophage-derived apoptotic vesicles regulate fate commitment of mesenchymal stem cells via miR155
Source: Stem Cell Res Ther. 2022 Jul 16;13:323. doi: 10.1186/s13287-022-03004-w (PMC9288680; doi:10.1186/s13287-022-03004-w)

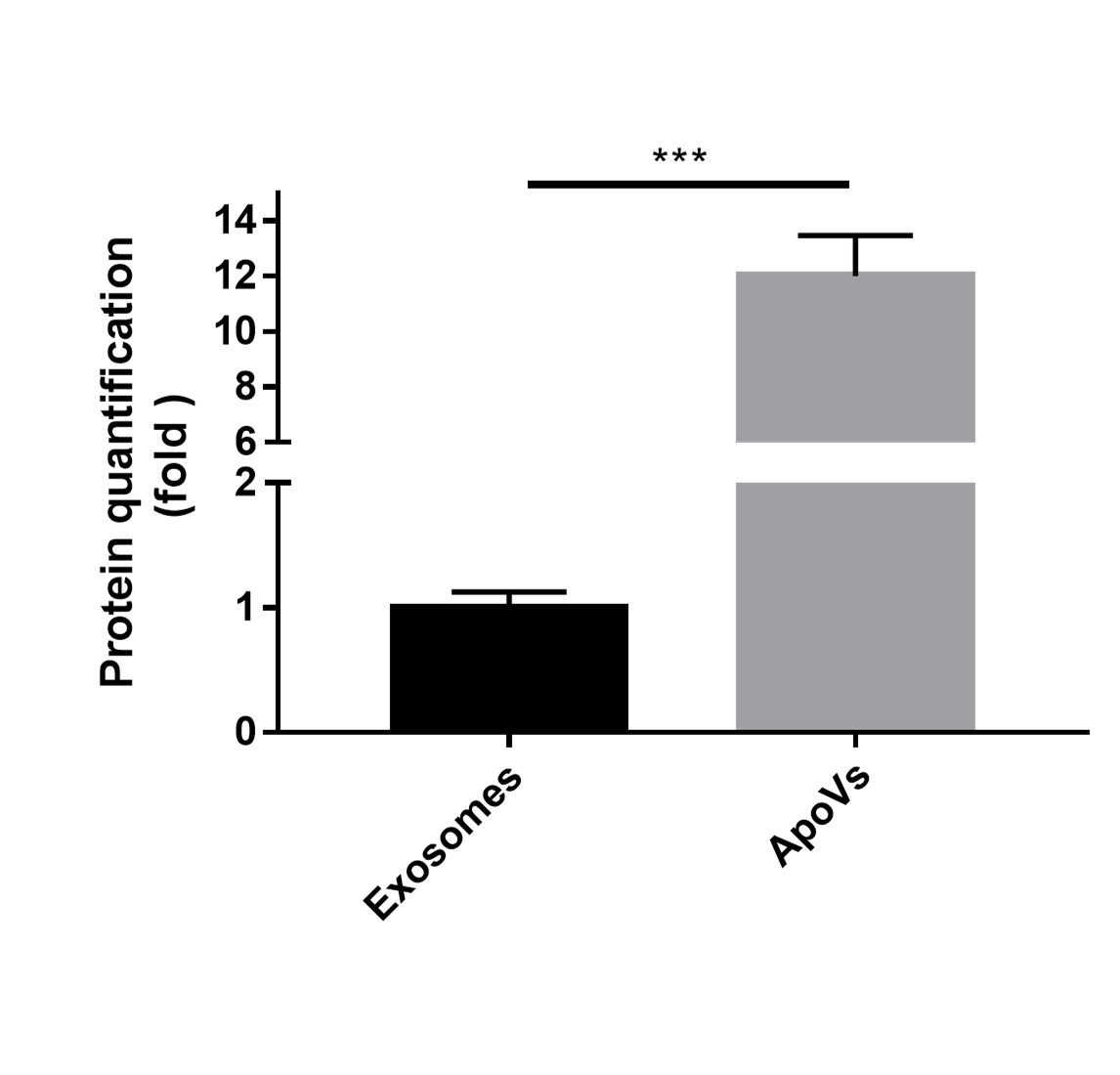

Supplement: Supplementary file 1 — Additional file 1: Fig.S1. Relative protein quantification of exosomes and apoVs. ***P < 0.001. [file 13287_2022_3004_MOESM1_ESM.tif]

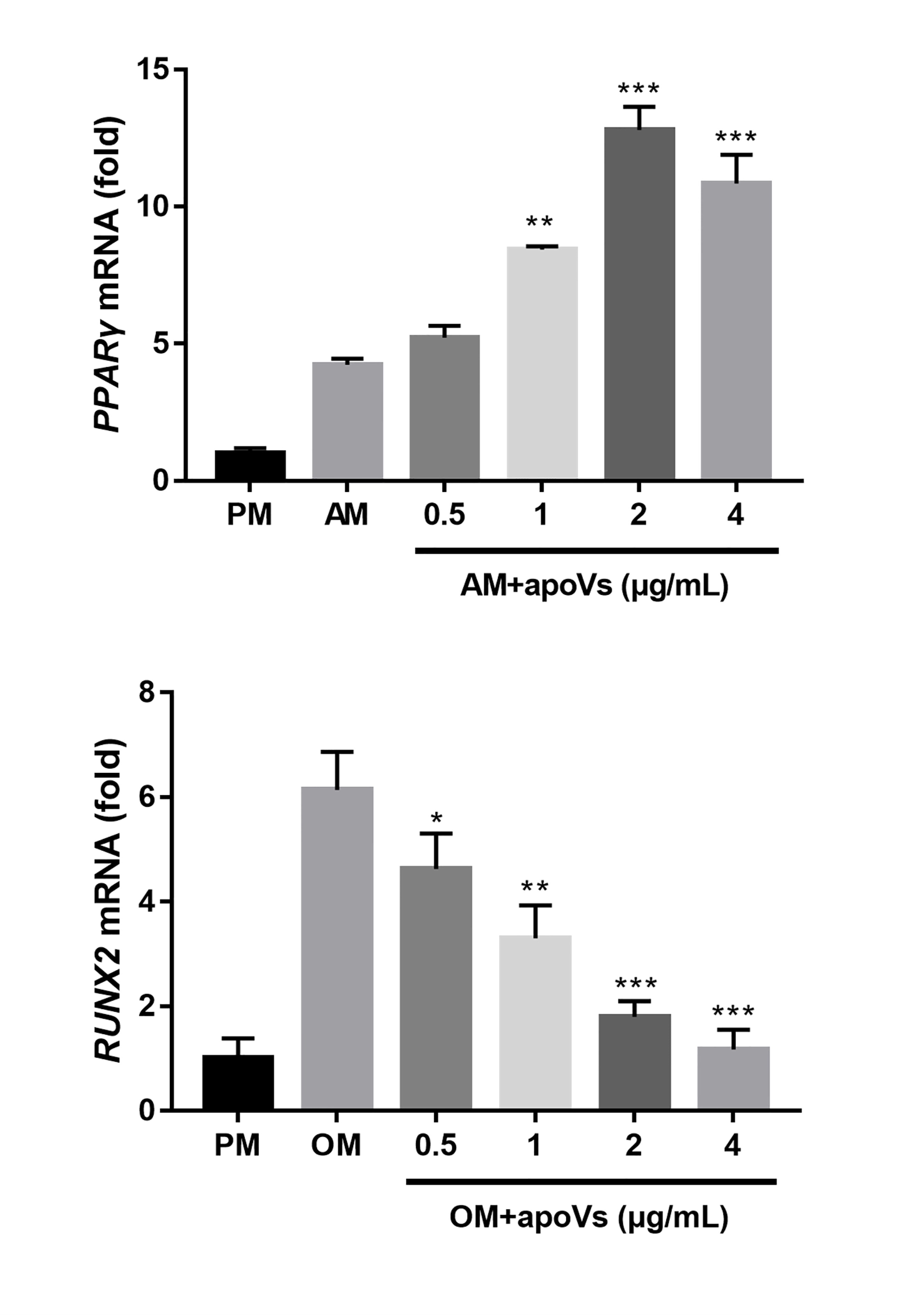

Supplement: Supplementary file 2 — Additional file 2: Fig. S2. The PPARγ and RUNX2 gene expression levels of MSCs treated with different concentration of macrophage-derived apoVs. *P < 0.05, **P < 0.01, ***P < 0.001 compared with AM or OM. [file 13287_2022_3004_MOESM2_ESM.tif]

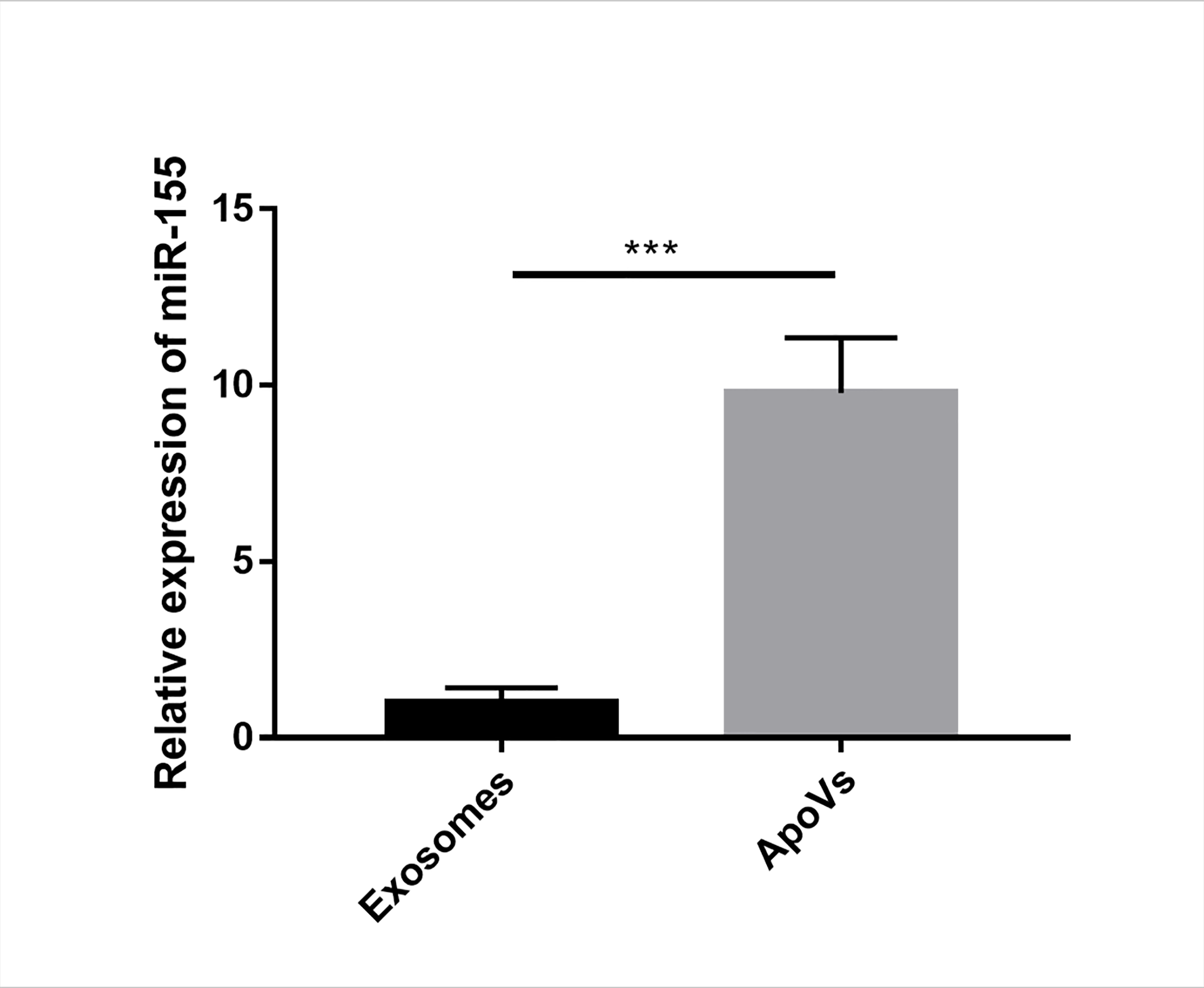

Supplement: Supplementary file 3 — Additional file 3: Fig. S3. Relative expression of miR155 in the macrophage-derived exosomes and apoVs. ***P < 0.001. [file 13287_2022_3004_MOESM3_ESM.tif]

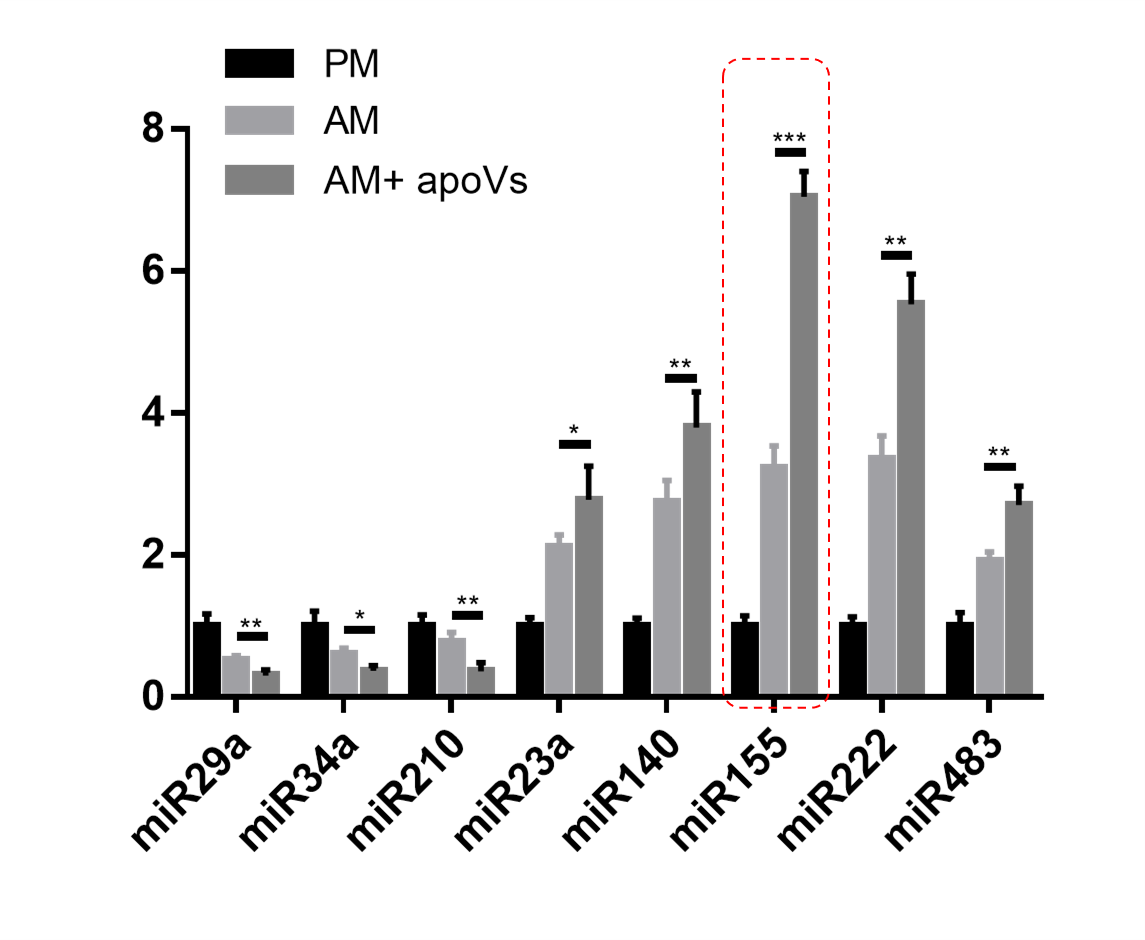

Supplement: Supplementary file 4 — Additional file 4: Fig. S4. Expression levels of different miRNAs in MSCs. *P < 0.05, **P < 0.01, ***P < 0.001 compared with AM. [file 13287_2022_3004_MOESM4_ESM.tif]

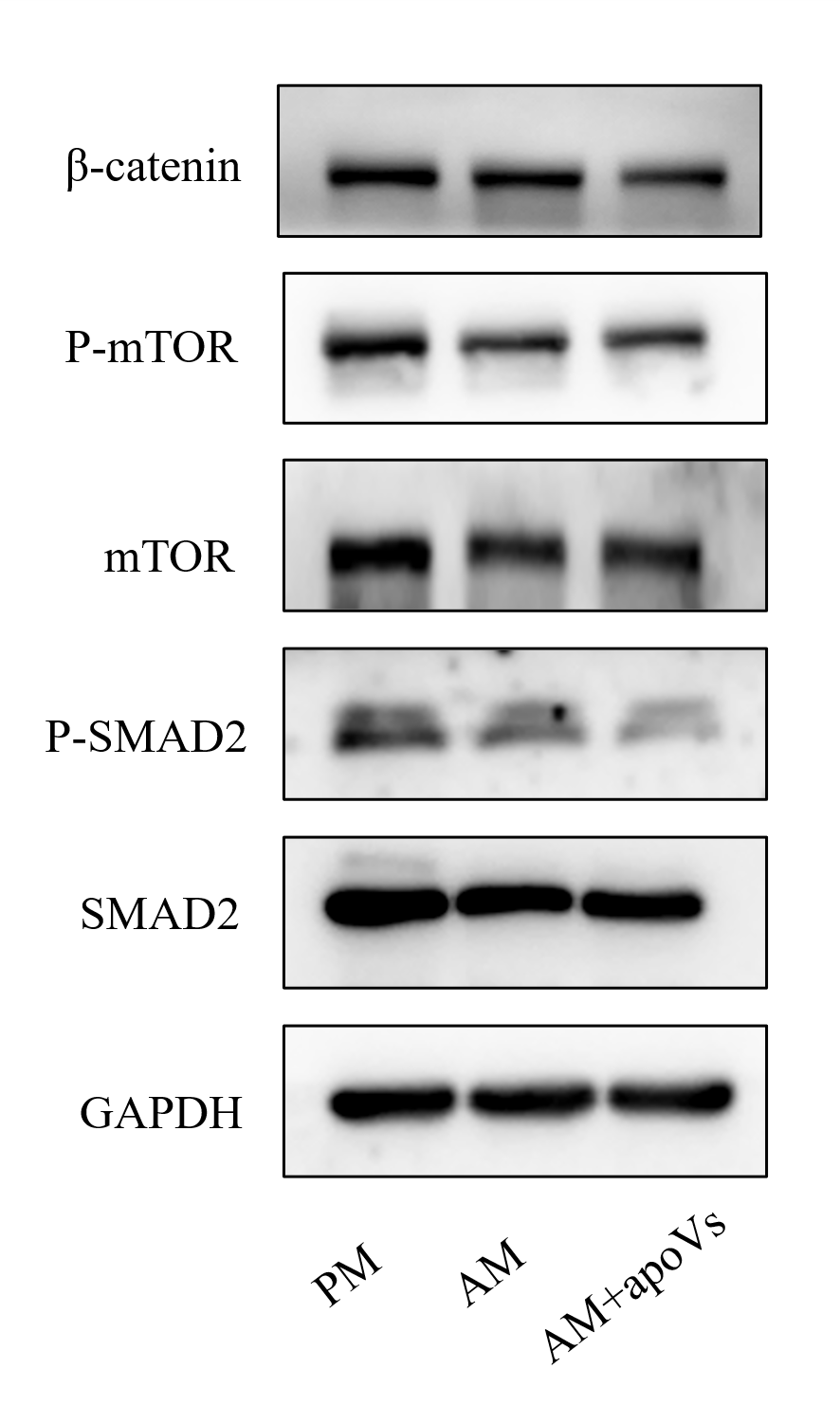

Supplement: Supplementary file 5 — Additional file 5: Fig. S5. Protein expression levels of different signaling pathways. [file 13287_2022_3004_MOESM5_ESM.tif]
